# *In Vitro* Assessment of Antiplasmodial Activity and Cytotoxicity of* Polyalthia longifolia* Leaf Extracts on* Plasmodium falciparum* Strain NF54

**DOI:** 10.1155/2019/6976298

**Published:** 2019-01-21

**Authors:** Bethel Kwansa-Bentum, Kojo Agyeman, Jeffrey Larbi-Akor, Claudia Anyigba, Regina Appiah-Opong

**Affiliations:** ^1^Department of Animal Biology and Conservation Science, School of Biological Sciences, College of Basic and Applied Sciences, University of Ghana, Legon, Ghana; ^2^Department of Clinical Pathology, Noguchi Memorial Institute for Medical Research, College of Health Sciences, University of Ghana, Legon, Ghana; ^3^Department of Biochemistry, Cell and Molecular Biology, College of Basic and Applied Sciences, University of Ghana, P.O. Box LG 54, Legon, Accra, Ghana; ^4^West African Centre for Cell Biology of Infectious Pathogens, Department of Biochemistry, Cell and Molecular Biology, College of Basic and Applied Sciences, University of Ghana, P.O. Box LG 54, Legon, Accra, Ghana

## Abstract

**Background:**

Malaria is one of the most important life-threatening infectious diseases in the tropics. In spite of the effectiveness of artemisinin-based combination therapy, reports on reduced sensitivity of the parasite to artemisinin in Cambodia and Thailand warrants screening for new potential antimalarial drugs for future use. Ghanaian herbalists claim that* Polyalthia longifolia* has antimalarial activity. Therefore, antiplasmodial activity, cytotoxic effects, and antioxidant and phytochemical properties of* P. longifolia* leaf extract were investigated in this study.

**Methodology/Principal Findings:**

Aqueous, 70% hydroethanolic and ethyl acetate leaf extracts were prepared using standard procedures. Antiplasmodial activity was assessed* in vitro* by using chloroquine-sensitive malaria parasite strain NF54. The SYBR® Green and tetrazolium-based calorimetric assays were used to measure parasite growth inhibition and cytotoxicity, respectively, after extract treatment. Total antioxidant activity was evaluated using a free radical scavenging assay. Results obtained showed that extracts protected red blood cells against* Plasmodium falciparum* mediated damage. Fifty percent inhibitory concentration (IC_50_) values were 24.0±1.08 *μ*g/ml, 22.5±0.12 *μ*g/ml, and 9.5±0.69 *μ*g/ml for aqueous, hydroethanolic, and ethyl acetate extracts, respectively. Flavonoids, tannins, and saponins were present in the hydroethanolic extract, whereas only the latter was observed in the aqueous extract. Aqueous and hydroethanolic extracts showed stronger antioxidant activities compared to the ethyl acetate extract.

**Conclusions/Significance:**

The extracts of* P. longifolia *have antiplasmodial properties and low toxicities to human red blood cells. The extracts could be developed as useful alternatives to antimalarial drugs. These results support claims of the herbalists that decoctions of* P. longifolia* are useful antimalarial agents.

## 1. Introduction

Human malaria is a parasitic disease caused by protozoan parasites* Plasmodium falciparum*,* Plasmodium vivax*,* Plasmodium malariae*,* Plasmodium ovale *[1],* Plasmodium knowlesi *[2], and* Plasmodium cynomolgi* [3]. The parasite is transmitted from human to human through the bite of an infective* Anopheles* mosquito. Currently, artemisinin-based combination drugs are used as first-line treatment of uncomplicated* P. falciparum* malaria [4]. Antimalarial drug resistance has emerged as one of the greatest challenges facing malaria control.* Plasmodium falciparum* resistance to artemisinins has already been detected in Asia [5–7]. The spread of drug resistant parasites and the limited number of effective drugs for treatment warrant the search for new antimalarial drugs.

It is estimated that there are 2.5 million species of higher plants throughout the world, most of which are yet to be exploited for their pharmacological activities [8]. Studies have shown that the use of herbal preparations in the treatment of malaria is rife in Ghana [9]. The flowering plant* Polyalthia longifolia* commonly called False Ashoka, Indian Mast tree, or the Buddha tree belongs to the family Annonaceae [10].* Polyalthia longifolia *is a lofty evergreen tree, native to India and Sri Lanka, and has been introduced in gardens of many tropical countries across the world including Ghana [10].* Polyalthia* is derived from a combination of Greek words meaning “many cures” with reference to the medicinal properties of the plant, whereas the Latin word* Longifolia* refers to the length of its leaves [11].

*Polyalthia longifolia* is used traditionally for the treatment of fever, skin diseases, diabetes, hypertension, and helminthiasis. In Ghana, decoctions of* P. longifolia* are widely used by native doctors for the treatment of malaria and fever. Phytochemical studies on* P*.* longifolia* have been carried out since 1980s and have often resulted in the isolation of diterpenoids and alkaloids [10]. Factors such as methods and solvents used for extraction may influence identification of phytochemicals in extracts. Studies on malaria have been carried out using several standard laboratory strains of the* P. falciparum* parasite including the chloroquine-sensitive (3D7) strain and the chloroquine-resistant (Dd2) strain. The use of* P. falciparum* standard laboratory chloroquine sensitive strain NF54 in* in vitro* studies has been recommended for better results due to higher reproducibility in* in vitro* cultures [12].

The aims of the study reported in this paper were to carry out phytochemical studies to assess the chemical components responsible for the antimalarial activities of the plant extracts. The antiplasmodial activity of* P. longifolia* leaf extracts on the NF54 parasite strain was evaluated using the SYBR Green assay. Fifty percent inhibitory concentration (IC_50_) values for the different leaf extracts were determined. Cell viability assay was performed to assess the cytotoxic effect of the extracts on human erythrocytes, using the tetrazolium-based colorimetric (MTT) assay. The leaf extracts were also screened for the presence of alkaloids, saponins, tannins, terpenoids, and flavonoids. Antioxidant activities were evaluated using the free radical scavenging assay and glutathione contents were also determined.

## 2. Materials and Methods

### 2.1. Plant Collection and Authentication

Fresh samples of* P. longifolia* leaves were collected from the University of Ghana main campus, Legon, Ghana. These were authenticated at the Centre for Plant Medicine Research herbarium, Mampong-Akuapem, Ghana, where voucher specimens (numbered 4061, 4062, and 4063) have been deposited.

### 2.2. Preparation of Plant Extracts

The leaves of* P. longifolia* were air-dried and pulverized with a blender. Twenty grams of the pulverised leaf sample was extracted with 200 ml of 70% ethanol and absolute ethyl acetate, separately. The process was repeated in order to increase the yield of the extracted samples. The extracts were then filtered through cotton wool-stuffed funnels. The filtrates were then concentrated by rotary vacuum evaporation under reduced pressure at −50°C. The aqueous portion were then frozen at −20°C and freeze-dried to obtain solid residue. Aqueous extraction was done by heating 20 g of the leaf samples in 200 ml of distilled water at 80°C for an hour. The extract was cooled and the supernatant separated using a cotton wool-stuffed funnel. The extraction procedure was repeated using the pellet to increase the yield. The supernatants were pooled, frozen at −20°C, and freeze-dried to obtain a dry powdered extract.

### 2.3. Parasite Cultivation

The antiplasmodial activity of leaf extracts was screened against chloroquine-sensitive* P. falciparum* NF54 strain obtained from continuous cultures. The parasites were cultured in human O^Rh+^ red blood cells according to the method of Trager and Jensen [14], using RPMI 1640 medium supplemented with 0.5% AlbuMAX II with hypoxanthine and buffered with 0.4% sodium bicarbonate (NaHCO_3_) and 0.72% N-2-hydroxyethylpiperazine-N-2-ethanesulfonic acid (HEPES). All the chemicals and reagents used for culturing were purchased from Sigma Chemical Company (St. Louis MO, USA) and Gibco BRL Life Technologies (Paisley, Scotland).

### 2.4. Antiplasmodial Activity of the Plant Extracts

The antiplasmodial activity of the extracts was assessed on cultured* P. falciparum* chloroquine-sensitive NF54 strain using the SYBR Green assay [15]. The extracts were weighed and dissolved in absolute methanol to obtain a stock solution of 100 mg/ml. The solution was then diluted with complete parasite media to a final working concentration of 10,000 *μ*g/ml (10 mg/ml). The stock solutions were filter-sterilised through a 0.2 *μ*m Millipore filters. Chloroquine was used as positive control. Two-fold serial dilutions of drugs (extracts) were performed to generate five concentrations for treatment of parasitized cells* in vitro*. One hundred microliters of* P. falciparum* malaria parasite culture suspension of NF54 (synchronized with 5% sorbitol to ring stage) was aliquoted into the wells of the pre-treated 96-well microtitre plate to a final haematocrit of 2% and parasitemia of 0.5%. Wells containing no drug but culture at the same parasitemia and haematocrit (with vehicle, i.e., distilled water) were included on each plate as negative control. Control wells also contained the vehicle and unparasitized red blood cells. Using the candle jar method, the plate was then placed in a humidified chamber in an incubator at 37°C for 72 h.

Evaluation of the outcome of the* in vitro *drug test was done by the SYBR Green method previously described [15]. Briefly, after 72 h of incubation of the parasite with the extract, 100 *μ*l malaria SYBR Green 1 fluorescent (MSF) lysis buffer (containing 20 mM Tris-Cl (pH 7.5), 5 mM EDTA, 0.008% Saponin, 0.08% Triton-X 100), and SYBR Green were added to each well and mixed thoroughly. The plate was covered with aluminium foil and incubated at room temperature in the dark for at least 3 h. The SYBR Green fluorescence was read on a multiwell plate reader (Tecan Infinite M200, Austria) at excitation and emission wavelengths set at 497 nm and 520 nm, respectively. The experiments were performed in triplicate and each repeated at least once. The concentrations at which 50% inhibition of growth was obtained (IC_50_ values) were determined by plotting the concentration of extract on* x*-axis against the percentage of inhibition on* y*-axis with dose response curves. Tables 1 and 2 contain two categories of antiplasmodial activities of plant extracts.

### 2.5. Screening of Plant Extracts for Cytotoxic Effects

A modified version of the tetrazolium-based colorimetric assay was used to screen the plant extracts for their toxicity to red blood cells [16, 17]. One hundred microliters of each of the crude and aqueous extracts, with concentration ranging from 1.23 *μ*g/ml to 300 *μ*g/ml, was placed in separate wells of a 96-well microtitre plate in duplicate. Subsequently, 100 *μ*l of uninfected red blood cells was added to each well. The contributions of plant extracts, culture medium, and uninfected red blood cells to the optical densities were excluded by setting up control experiments for each of the parameters separately alongside the main experiments. The plates were then incubated at 37°C for 3 days in a humidified incubator at 5% O_2_ and CO_2_ before 20 *μ*l of 7.5 mg/ml MTT (in phosphate buffered saline) solution was added to each well and the plate incubated again for 2 h. Aliquots of culture media (150 *μ*l) were removed from each well and discarded after incubation and 200 *μ*l of Triton X-100 in acidified isopropanol was added to each well to dissolve any formazan formed. The plates were then kept at room temperature in the dark for 24 h and the optical densities of the wells were read at 570 nm on the plate reader. The concentrations at which 50% cytotoxic effect occurred (CC_50_ values) were then determined by plotting concentration of extract on* x*-axis and percentage of cell viability on* y*-axis with dose-response curves (using Microsoft Excel 2016 software). The CC_50_ values were compared with standard values for activity and their biological substances as described [11, 13].

### 2.6. Determination of Antioxidant Activity of Extracts by Free Radical Scavenging Activity

The antioxidant activity of the extracts was assessed using the free radical scavenging activity as described [18]. The freeze-dried extracts were dissolved in methanol (final concentration range 0.015 – 0.5 mg/ml) and incubated with methanolic DPPH in the dark at room temperature for 20 min. Absorbance was measured using a microplate reader (Tecan Infinite M200 Pro, Switzerland) at a wavelength of 517 nm. Butylated hydroxytoluene was used as positive control. Triplicate experiments were performed. Mean percentage antioxidant (scavenging) activity was plotted against extract concentrations, and the effective concentrations at which 50% antioxidant activity (EC_50_) occurred were extrapolated from the graphs.

### 2.7. Determination of Reduced Glutathione Content

The reduced glutathione (GSH) content of the extracts was determined using a described procedure [19, 20]. The reaction mixture was comprised of GSH buffer (pH 8) and extracts or GSH standard and 0.0075 mM O-phthalaldehyde solution. The mixture was incubated in the dark at room temperature for 15 min and absorbance was read at a wavelength of 412 nm. Triplicate experiments were performed. The GSH content of each extract was calculated from a regression equation derived from a GSH standard calibration curve.

### 2.8. Phytochemical Screening

The three leaf extracts were screened for the presence of selected phytochemical constituents using standard procedures as previously described [21–23]. The extracts were dissolved in appropriate solvents as described below, depending on the method used. Positive controls were used in each case.

#### 2.8.1. Saponins

For saponins, 1 ml of the extracts was dissolved in distilled water. This was vigorously shaken for one minute. A stable persistent froth indicated the presence of saponins.

#### 2.8.2. Alkaloids

For alkaloids, 1 ml of the extracts was dissolved in distilled water and two millilitres of Wagner's reagent (Iodo-potassium iodide) was added. Formation of a reddish-brown precipitate indicated the presence of alkaloids. Quinidine was used as positive control.

#### 2.8.3. Terpenoids

For terpenoids, 1 ml of the extracts was dissolved in distilled water. One millilitre of chloroform was added after which one millilitre concentrated H_2_SO_4_ was gently added. The formation of a reddish-brown colour at interface indicated the presence of terpenoids. Ursolic acid was used as positive control.

#### 2.8.4. Tannins

For tannins, 1 ml of the extracts was dissolved in distilled water. This was boiled for about 10 min after which 3 drops of 0.1% FeCl_3_ was added to the supernatant. A brownish-green or blue-black colour indicated the presence of tannins. Gallic acid was used as positive control.

#### 2.8.5. Flavonoids

For flavonoids, one millilitre of dilute ammonia solution was added to four millilitres of the aqueous filtrate of the plant extracts, followed by the addition of few drops of concentrated H_2_SO_4_. A yellow coloration indicated the presence of flavonoids. Quercetin was used as positive control.

### 2.9. Data Analyses

Experiments were performed in triplicates and were repeated at least once. Data was presented as mean ± standard deviation (SD). Microsoft Excel 2016 and GraphPad Prism 5.0 version (GraphPad Prism software Inc., San Diego CA) software were used in plotting the graphs. The IC_50_, CC_50_, and EC_50_ values were obtained from dose-response curves also using the above software. Statistical analysis was performed using the Student* t*-test; *ρ* < 0.05 was considered statistically significant.

### 2.10. Ethical Clearance

The study was reviewed and approved by the Scientific and Technical Committee and the Institutional Review Board (NMIMR-IRB CPN 001/12-13 revd 2017), both of the Noguchi Memorial Institute for Medical Research (NMIMR), College of Health Sciences, University of Ghana, Legon. Written informed consent was sought from volunteers who donated the blood samples that were used for the* in vitro* culturing of the parasite.

## 3. Results

### 3.1. Antiplasmodial Activity of* Polyalthia longifolia* Leaf Extracts

**Figure 1:**
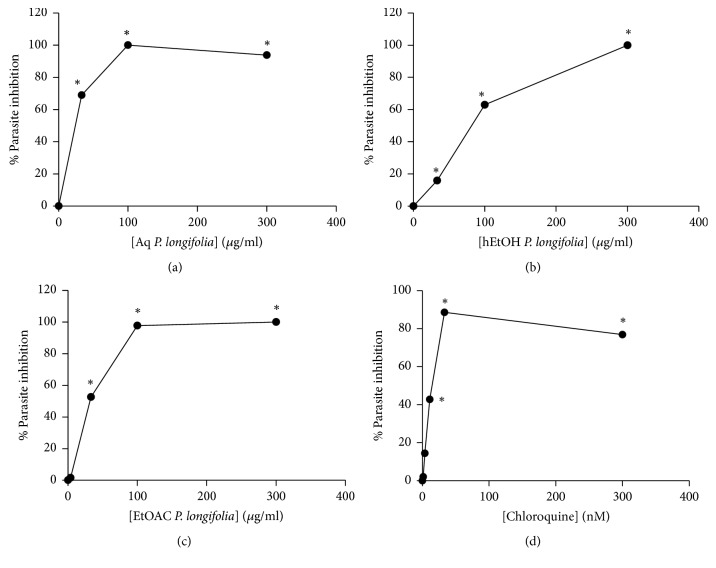
**Dose-response curves of the plant extracts and chloroquine**. The percentage parasite inhibition drawn against the concentrations of the leaf extracts [aqueous extract ((a) Aq.* P. longifolia*), 70% ethanol ((b) hEtOH* P. longifolia*), ethyl acetate ((c) EtOAc* P. longifolia*)], and (d) chloroquine (CHQ). Data represent means for three experiments, with “*∗*” indicating statistically significant difference (p<0.05) from the control (uninfected RBCs).

The inhibition patterns of the leaf extracts against the NF54 strain of* P*.* falciparum* are shown in Figure 1. Among the three leaf extracts, ethyl acetate extract showed the strongest antiplasmodial activity with IC_50_ value of 9.5 *μ*g/ml (Table 3). The selectivity indices of all the leaf extracts tested were greater than 4 (Table 3).

### 3.2. Erythrocyte Cytotoxicity of* Polyalthia longifolia* Leaf Extracts

**Figure 2:**
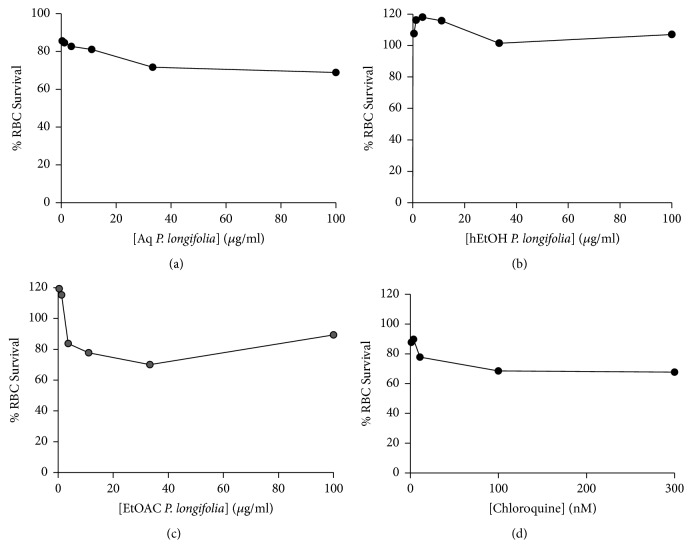
**Red blood cell survival after exposure to the leaf extracts and chloroquine**. Leaf extracts [aqueous extract ((a) Aq.* P. longifolia*), 70% ethanol ((b) hEtOH* P. longifolia*), ethyl acetate ((c) EtOAc* P. longifolia*)], and (d) chloroquine. Data represent means for three experiments, with “*∗*” indicating statistically significant difference (p<0.05) from the control (untreated RBCs).

Results from the survival rate of human red blood cell after they were exposed to the leaf extracts are shown in Figure 2. The leaf extracts exhibited similar trends having higher percent cell survival than the positive control.

Table 3 shows that all the extracts had good selectivity towards the malaria parasites since all the selectivity indices were greater than 2.

### 3.3. Phytochemical Analyses of* Polyalthia longifolia* Leaf Extracts

Five qualitative chemical tests were performed on the three leaf extracts to determine the presence of saponins, alkaloids, terpenoids, tannins, and flavonoids. The results showed the presence of saponins, tannins, and flavonoids mostly in the hydroethanolic extracts (Table 4).

### 3.4. Antioxidant Activity of* Polyalthia longifolia *Extracts

**Figure 3:**
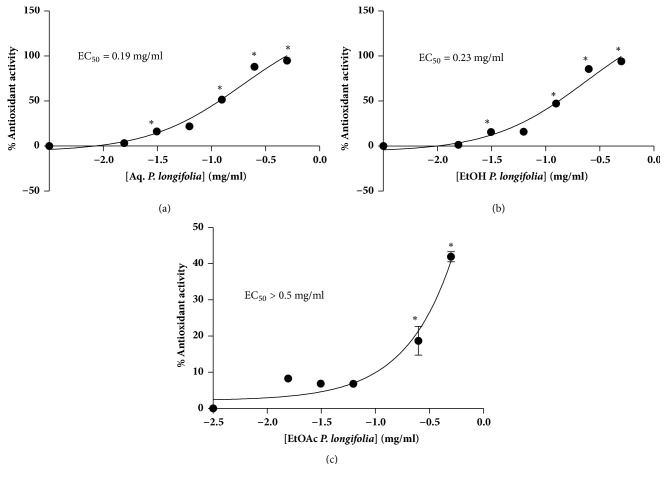
**Antioxidant properties of* Polyalthia longifolia *leaf extracts**. Leaf extracts [aqueous extract ((a) Aq.* P. longifolia*), 70% ethanol ((b) EtOH* P. longifolia*), and ethyl acetate ((c) EtOAc* P. longifolia*)]. Data represent means for three experiments, with “*∗*” indicating statistically significant difference (p<0.05) from the negative control (methanol treated).

**Figure 4:**
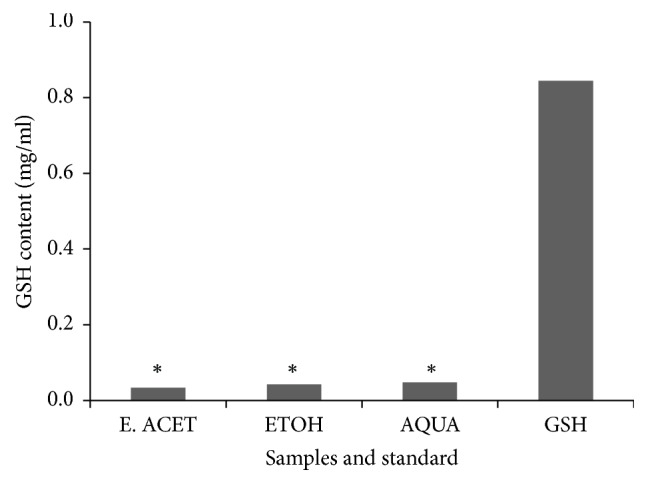
**Concentrations of reduced glutathione of* Polyalthia longifolia *leaf extracts**. The leaf extracts: aqueous extract (AQUA), 70% ethanol (ETOH), ethyl acetate (E. ACET), and the control (GSH). Data represent means for three experiments, with “*∗*” indicating statistically significant difference (p<0.05) from the control (GSH).

Total antioxidant activities of the extracts are shown in Figure 3. The aqueous extracts have the strongest activity with EC_50_ value of 0.19 mg/ml. This was however similar to that of the hydroethanolic extract (0.23 mg/ml). A weak activity was observed with the ethyl acetate extract. Similarly, reduced GSH levels of the aqueous and hydroethanolic extract were higher than the level in the ethyl acetate extract (Figure 4).

## 4. Discussion

Over the years, plant medicine research has gradually increased as a means of finding promising herbs and novel chemical compounds to fight diseases including malaria [24]. Traditional medicine is one of the most patronized means of treatment in Ghana [25, 26]. Traditional Medicine Practitioners in Ghana use plants such as* P. longifolia* to manage malaria. In the present study, aqueous, hydroethanolic, and ethyl acetate extracts of* P. longifolia* were evaluated for antiplasmodial activity using the SYBR Green assay. SYBR Green is an asymmetrical cyanine dye, which binds to any double-stranded DNA, including the DNA intrinsically present in whole blood samples, preferring G and C base pairs [27]. The assay was used to quantify the parasite DNA present in the wells of the assay plate, with each of the different concentrations of the extracts after they were incubated. Graphs of dose-response inhibition showed that each of the extracts of* P. longifolia* has antiplasmodial activities, based on the IC_50_ values obtained.

Plant extracts can be classified either as good or as poor potential drugs based on their IC_50_ values [13]. More importantly, effectiveness of plant extracts in terms of whether the extracts have inherent active biological substance has been grouped into four categories based on their* in vitro* antiplasmodial IC_50_ values [11]. A study into the activity of herbal medicines on 3D7 strain of* P. falciparum* gametocytes showed IC_50_ values ranging from 1.41 to 82.59 ng/ml [9]. Findings in that same study suggest that culturing the asexual stage of the parasite in the presence of low doses of some herbal products increased the commitment signal for gametocyte production [9]. In another study on drug sensitivity assessment of field isolates, IC_50_ values for artesunate and chloroquine ranged within 0.2–3.6 nM and 2–200 nM, respectively [28]. The IC_50_ values for the reference strain FCR3/FMG used as control in that study were 3.25 and 20.71 nM for artesunate and chloroquine, respectively [28]. Comparing the findings of the above studies to that of the present one, both the aqueous and hydroethanolic extracts of* P. longifolia *showed good antiplasmodial activity, whereas the ethyl acetate extract exhibited a promising antiplasmodial activity. The MTT assay was used to determine the selective action of the extract as described [16] instead of the SYBR Green assay and because mature red blood cells used for the study lack nucleic acids the SYBR Green assay involves binding of the SYBR Green dye to double stranded DNA which becomes fluorescent when bound and can be detected [27]. On the other hand, the principle of the MTT assay is the ability of the cells being tested to convert yellowish tetrazolium into formazan a dark purple compound by viable enzymes in the red blood cells. The specific enzyme(s) involved in the conversion is not yet known [16]. The conversion would only be possible if only the extracts they are treated with do not kill the cells. The ability of the red blood cells to undergo the conversion therefore is linked to the plant extract's intrinsic components which protects the red blood cells against cell-mediated damage. The results from the tetrazolium-based colorimetric (MTT) assay suggest that the plant extracts have intrinsic biological components that enable them to protect the human red blood cells against* P. falciparum* mediated damage [17].

The CC_50_ values of the extracts could not be determined in the present study, indicating the weak cytotoxicity potential of the extracts. Even though similar trends were observed, hydroethanolic extract showed the highest percent cell survival values as chloroquine had the lowest percent cell survival values. This suggests that although both drugs exhibit low toxicity to red blood cells, chloroquine is more toxic to red blood cells as compared to the hydroethanolic extract. All the extracts tested had good selectivity indices (>2), suggesting good therapeutic potentials. The presence of phytochemical and bioactive compounds in plants is responsible for the medicinal values and is indicative of their many possible therapeutic uses [29]. In the present study, saponins, tannins, and flavonoids were the presence in the hydroethanolic extract. Saponins were identified in the aqueous extract whereas none of the phytochemical investigated was found in the ethyl acetate extract. The phytochemicals present in the extracts confirmed what was reported by Kumari et al. [11] that* P. longifolia* leaves contain saponins, tannins, and flavonoids. Ethyl acetate leaf extract of* P. longifolia* does not contain saponins and terpenoids [11]. However, the ethyl acetate extract was observed to exhibit the most potent activity. This suggests that other phytochemicals which were not tested for in the present study may be responsible for the promising antiplasmodial activity exhibited by the ethyl acetate extract. These results as well as those of other malaria research works are consistent with claims of Ghanaian Traditional Medicine Practitioners that some plants including* P. longifolia* have good antimalarial therapeutic effect [11, 17, 30]. From our results, the selectivity of all the extracts is greater than four. This suggests that the extracts have good selectivity for the malaria parasites making them good drug candidates because the SI values were greater than 2 [31].

The* P. falciparum* infected red blood cells are constantly exposed to oxidative stress due to reactive oxygen and nitrogen species produced by the host immune system during various processes [32]. GSH is the body's main endogenous antioxidant source for mopping up free radical. Thus, it is critical for maintaining the homeostasis particularly in disease conditions. Therefore, antioxidant activity of the extracts was evaluated using the free radical scavenging assay and glutathione contents of the extracts were also determined. Infected erythrocytes are constantly under oxidative stress from both exogenous and endogenous processes. Therefore, in diseases such as malaria which highly expose erythrocytes to oxidative stress it is helpful to have antimalarial agents which also have antioxidant activities. Hence total antioxidant and reduced glutathione levels of the extracts were evaluated. All the extracts contained reduced glutathione. In general, none of the plant extracts was overly toxic to human red blood cells in culture. Due to the low toxicity levels, the median cytotoxic concentration (CC_50_) could not be determined for any of the three extracts. This suggests that all the extracts are good and potential sources of future antimalarial drugs.

## 5. Conclusions

The results of this study lend support to earlier reports that* P. longifolia* has good antiplasmodial activity. Aqueous, hydroethanolic, and ethyl acetate extracts of* P. longifolia* have good* in vitro* antiplasmodial activity against chloroquine-sensitive* P. falciparum* NF54 strain. The aqueous, hydroethanolic, and ethyl acetate extracts have considerably low toxicities to human red blood cells. The phytochemicals present in the extracts include saponins, tannins, and flavonoids. These may be responsible for the antiplasmodial properties exhibited by the extracts. The results of this study provide scientific basis to claims by herbalists that decoctions of* P. longifolia* are useful antimalarial remedies. It is therefore recommended that* in vivo *efficacy studies be carried out using the extracts. Further studies should also be conducted to isolate compounds present in the extracts and screen them for antiplasmodial activity.* In vivo* toxicity studies must also be performed on the extracts using animal models.

## Figures and Tables

**Table 1:** Categorisation of activities of plant extracts against *Plasmodium falciparum*.

**IC**_**50**_ ** value (** ***μ*** **g/ml)**	**Category of activity**
< 10	Promising
10 – 20	Moderate
20 – 40	Good
40 – 70	Marginally potent
> 70	Poor

Source [13].

**Table 2:** Categorisation of biological substance of plant extracts based on antiplasmodial activity.

**IC**_**50**_ ** value (** ***μ*** **g/mL)**	**Category of biological substances**
< 5	Very active
5 – 50	Active
50 – 100	Weakly active
> 100	Inactive

Source [11].

**Table 3:** Antiplasmodial and cytotoxic effects of *Polyalthia longifolia* leaf extracts and chloroquine.

**Plant extract**	**I** **C**_50_ ± **SD (*μ*g/ml)**	**C** **C**_50_ ** (** ***μ*** **g/ml)**	**Selectivity Indices**
Aqueous	24.00 ± 1.08*∗∗*	> 100	> 4.17*∗∗*
70% Ethanol	22.46 ± 0.12*∗∗*	> 100	> 4.45*∗∗*
Ethyl acetate	9.50 ± 0.69*∗∗*	> 100	> 10.53*∗∗*
Chloroquine*∗*	4.71 ± 0.02	> 95.96	> 20.38

*∗*Chloroquine was used as a positive control.

Data represent means for three experiments ± SD (standard deviation). The symbol “*∗∗*” indicates statistically significant difference (*ρ* < 0.05) from the positive control.

**Table 4:** Phytochemical content of plant extracts.

Phytochemical	Aqueous Extract	Hydro-Ethanolic Extract	Ethyl Acetate Extract
Saponins	+	+	-
Alkaloids	-	-	-
Terpenoids	-	-	-
Tannins	-	+	-
Flavonoids	-	+	-

^+^Present.

^–^Absent.

## Data Availability

The data used to support the findings of this study are included within the article.

## References

[1] Ong'echa J. M., Keller C. C., Were T. (2006). Parasitemia, anemia, and malarial anemia in infants and young children in a rural holoendemic Plasmodium falciparum transmission area. *The American Journal of Tropical Medicine and Hygiene*.

[2] Singh B., Daneshvar C. (2013). Human infections and detection of Plasmodium knowlesi. *Clinical Microbiology Reviews*.

[3] Ta H. T., Hisam S., Lanza M., Jiram A. I., Ismail N., Rubio J. M. (2014). First case of a naturally acquired human infection with Plasmodium cynomolgi. *Malaria Journal*.

[4] World Health Organization (2015). *Guidelines for the treatment of Malaria*.

[5] Hengling Y., Dequan L., Yaming Y. (2003). Changes in susceptibility of Plasmodium falciparum to artesunate in vitro in Yunnan Province, China. *Transactions of the Royal Society of Tropical Medicine and Hygiene*.

[6] Alker A. P., Lim P., Sem R. (2007). PFMDR1 and in vivo resistance to artesunate-mefloquine in falciparum malaria on the Cambodian-Thai border. *The American Journal of Tropical Medicine and Hygiene*.

[7] Dondorp A. M., Nosten F., Yi P. (2009). Artemisinin resistance in Plasmodium falciparum malaria. *The New England Journal of Medicine*.

[8] Bobby M. N., Wesely E. G., Johnson M. A. (2012). In vitro anti– bacterial activity of leaves extracts of Albizia lebbeck Benth against some selected pathogens. *Asian Pacific Journal of Tropical Biomedicine*.

[9] Amoah L. E., Kakaney C., Kwansa-Bentum B., Kusi K. A., Lanz-Mendoza H. (2015). Activity of herbal medicines on plasmodium falciparum gametocytes: implications for malaria transmission in Ghana. *PLoS ONE*.

[10] Jothy S. L., Choong Y. S., Saravanan D. (2013). Polyalthia longifolia Sonn: An ancient remedy to explore for novel therapeutic agents. *Research Journal of Pharmaceutical, Biological and Chemical Sciences*.

[11] Kumari S. D., Satish P. V., Somaiah K., Rekha S. N., Brahmam P., Sunita K. (2016). Antimalarial activity of Polyalthia longifolia (False Ashoka) against chloroquine sensitive Plasmodium falciparum 3D7 strain. *World Journal of Pharmaceutical Sciences*.

[12] Roncalés M., Vidal-Mas J., Leroy D., Herreros E. (2013). Comparison and optimization of different methods for the in vitro production of Plasmodium falciparum gametocytes. *Journal of Parasitology Research*.

[13] Kamaraj C., Kaushik N. K., Mohanakrishnan D. (2012). Antiplasmodial potential of medicinal plant extracts from Malaiyur and Javadhu hills of South India. *Journal of Parasitology Research*.

[14] Trager W., Jensen J. B. (1980). *Cultivation of Erythrocytic And Exoerythrocytic Stages of Plasmodium in Malaria*.

[15] Johnson J. D., Dennull R. A., Gerena L., Lopez-Sanchez M., Roncal N. E., Waters N. C. (2007). Assessment and continued validation of the malaria SYBR Green I-based fluorescence assay for use in malaria drug screening. *Antimicrobial Agents and Chemotherapy*.

[16] Ayisi N. K., Appiah-Opong R., Gyan B., Bugyei K., Ekuban F. (2011). Plasmodium falciparum: Assessment of selectivity of action of chloroquine, Alchornea cordifolia, Ficus polita and other drugs by tetrazolium-based colorimetric assay. *Malaria Research and Treatment*.

[17] Appiah-Opong R., Nyarko A. K., Dodoo D., Gyang F. N., Koram K. A., Ayisi N. K. (2011). Antiplasmodial activity of extracts of Tridax procumbens and Phyllanthus amarus in in vitro Plasmodium falciparum culture systems. *Ghana Medical Journal*.

[18] Appiah-Opong R., Ofori-Attah E., Agordzo E., Nyarko AK., Ankrah N-A. (2015). Inhibition of aflatoxin B1-8, 9-epoxide formation by selected Ghanaian vegetables. *Journal of the Ghana Science Association*.

[19] Hissin P. J., Hilf R. A. (1976). *A Fluorometric Method for Determination of Oxidized and Reduced Glutathione in Tissues*.

[20] Acheampong F., Larbie C., Appiah-Opong R., Arthur F., Tuffour I. (2015). In vitro Antioxidant and Anticancer Properties of Hydroethanolic Extracts and Fractions of Ageratum conyzoides. *European Journal of Medicinal Plants*.

[21] Sofowora A. (1993). *Medicinal Plants and Traditional Medicine in Africa*.

[22] Harborne A. J. (1998). *Phytochemical Methods: A Guide to Modern Techniques of Plant Analysis*.

[23] Njoku O. V., Obi C. (2009). Phytochemical constituents of some selected medicinal plants. *African Journal of Pure and Applied Chemistry*.

[24] Tilburt J. C., Kaptchuk T. J. (2008). Herbal medicine research and global health: An ethical analysis. *Bulletin of the World Health Organization*.

[25] Kwansa-Bentum B., Ayi I., Suzuki T. (2011). Administrative practices of health professionals and use of artesunate-amodiaquine by community members for treating uncomplicated malaria in southern Ghana: implications for artemisinin-based combination therapy deployment. *Tropical Medicine & International Health*.

[26] Gbedema S. Y., Adu F., Bayor M. T., Annan K., Boateng J. S. (2010). Enhancement of antibacterial activity of amoxicillin by some Ghanaian medicinal plant extracts. *International Journal of Pharmaceutical Sciences and Research*.

[27] Vossen M. G., Pferschy S., Chiba P., Noedl H. (2010). The SYBR green I malaria drug sensitivity assay: Performance in low parasitemia samples. *The American Journal of Tropical Medicine and Hygiene*.

[28] Kwansa-Bentum B., Ayi I., Suzuki T. (2011). Plasmodium falciparum isolates from southern Ghana exhibit polymorphisms in the SERCA-type PfATPase6 though sensitive to artesunate in vitro. *Malaria Journal*.

[29] Biapa P.-C. N., Agbor G. A., Oben J. E., Ngogang J. Y. (2007). Phytochemical studies and antioxidant properties of four medicinal plants used in Cameroon. *African Journal of Traditional, Complementary and Alternative Medicines*.

[30] Adika C., Aboagye-Antwi F., Appiah-Opong R., Duah N. O., Ayanful-Torgby R., Matrevi S. (2016). Assessment of the effects of natural cocoa extract on erythrocyte membrane and asexual erythrocytic stage of plasmodium falciparum. *Asian Journal of Ethnopharmacology and Medicinal Foods*.

[31] Badisa R. B., Darling-Reed S. F., Joseph P., Cooperwood J. S., Latinwo L. M., Goodman C. B. (2009). Selective cytotoxic activities of two novel synthetic drugs on human breast carcinoma MCF-7 cells. *Anticancer Reseach*.

[32] Bozdech Z., Ginsburg H. (2004). Antioxidant defense in plasmodium falciparum - data mining of the transcriptome. *Malaria Journal*.

